# Association between α-klotho levels and adults with COPD in the United States

**DOI:** 10.3389/fmed.2024.1361922

**Published:** 2024-07-18

**Authors:** Dan Yan

**Affiliations:** Department of Pulmonary and Critical Care Medicine, Jinhua Municipal Central Hospital, The Affiliated Jinhua Hospital, College of Medicine, Zhejiang University, Jinhua, China

**Keywords:** α-klotho, COPD, anti-aging, NHANES, chronic obstructive pulmonary disease

## Abstract

**Purpose:**

Chronic obstructive pulmonary disease (COPD) is accompanied by increased inflammation, persistent lung function decline, and extensive lung injury. Klotho, a well-known antiaging protein, has anti-inflammatory and antioxidative effects. However, the effects of klotho on COPD have yet to be thoroughly elucidated. This study examined the association among COPD adults and their α-klotho level.

**Patients and methods:**

Data were collected from the 2007 to 2012 National Health and Nutrition Examination Survey (NHANES). A total of 676 participants were analyzed and divided into COPD (*n* = 403) and non-COPD (*n* = 273) groups. The two groups were compared with respect to clinical characteristics. Logistic regression analysis and a generalized additive model were used to estimate the association between COPD incidence and serum α-klotho concentration. All COPD participants were stratified according to the levels of α-klotho (Q1: <687 pg./mL; Q2: 687–900 pg./mL; Q3: ≥900 pg./mL), and clinical characteristics were compared.

**Results:**

Non-COPD individuals had higher α-klotho levels than did COPD individuals (863.09 ± 267.13 vs. 817.51 ± 302.20, *p* < 0.05). Logistic regression analysis revealed that the Q2 and Q3 layers had a lower risk of COPD than did the Q1 layer, with odds ratios (ORs) of 0.73 (0.50, 0.99) for Q2 and 0.58 (0.41, 0.86) for Q3 (*p* < 0.001). The generalized additive model showed that the risk of COPD gradually decreased with increasing α-klotho concentration when the α-klotho concentration < 1,500 pg./mL, while the risk of COPD increased as the α-klotho concentration increased to ≥1,500 pg./mL. Compared with individuals in the Q2 or Q3 groups, individuals with COPD in the Q1 group were more likely to be current smokers, have lower levels of erythrocytes, and have higher levels of creatinine and leukocytes.

**Conclusion:**

Increased α-klotho levels were negatively correlated with the risk of COPD in participants over 40 years old with α-klotho <1,500 pg./mL. When α-klotho was ≥1,500 pg./mL, the risk of COPD increased as α-klotho levels increased. Pulmonary ventilation function and the number of hemocytes differed among COPD patients with different levels of α-klotho.

## Introduction

COPD, which warrants significant concern due to its high incidence and mortality, is characterized by progressive lung function decline and largely irreversible airflow obstruction (1). Globally, COPD burdens health care systems significantly according to the World Bank/World Health Organization. COPD is more common in adults over 40, with a prevalence of 6–10% (2). The prevalence of COPD is rampant in China, with 13.7% of people aged 40 and older suffering from it (3).

COPD is a lung condition that accelerates lung aging and is associated with an abnormal inflammatory response, which is chronic and damaging (4). Over the past decade, the close association between COPD and inflammation has become increasingly apparent (5). Many factors associated with COPD, such as dietary patterns and exposure to cigarette smoke, can significantly influence inflammation levels (6, 7). Moreover, COPD lungs exhibit many features of normal aging (oxidative stress, increased cellular senescence, and stem cell exhaustion); therefore, it has been suggested that COPD is characterized by accelerated aging (8, 9). Although lung aging is a natural process, inflammatory-oxidative stress can accelerate the cellular senescence of pulmonary epithelial cells (10). Additionally, aging and chronic inflammation share several underlying molecular and genetic mechanisms with inflammation in COPD (11). Therefore, we wondered whether some senescence-associated proteins might affect the development of COPD.

Klotho is a protein with anti-aging properties that involves endogenous antioxidants, senescence, and chronic inflammation (12). KLOTHO gene expression may be closely related to COPD. Klotho-deficient mice exhibit a remarkable number of important aging features, including a shortened lifespan, sarcopenia, and features of emphysema (increased air spaces, increased expiration time, and damaged alveolar walls) (12, 13). Some animal studies have suggested that the induction of klotho expression may reverse advanced age-associated emphysema (14, 15). Several clinical studies have demonstrated a decrease in KLOTHO gene expression in the airways of COPD patients compared to non-smokers (16, 17), as well as lower levels of KLOTHO gene expression in their alveolar macrophages (18). Moreover, research indicates a potential connection between serum α-klotho and the development of COPD. Kureya et al. identified a noteworthy positive association between serum α-klotho and irisin, a myokine implicated in the advantageous effects of physical activity (19), suggesting that α-klotho may play a role in modulating skeletal muscle function in COPD (20). Another study identified an inverse correlation between serum α-klotho and resistin (a signaling molecule associated with muscle strength) (21) following a six-month training program in COPD patients (22). In a recent study, systemic α-klotho levels correlated negatively with COPD prevalence (23). These findings indicate that serum α-klotho may be a potential therapeutic target for COPD. However, clinical studies investigating its relevance to systemic α-klotho levels are still lacking.

The purpose of this study was to examine the association between α-klotho and COPD using data from a public database of data collected from the US population between 2007 and 2012. Our research might have the potential to substantially enhance the early detection rates of COPD, thereby facilitating the prompt initiation of intervention strategies.

## Materials and methods

### Data source

**Figure 1:**
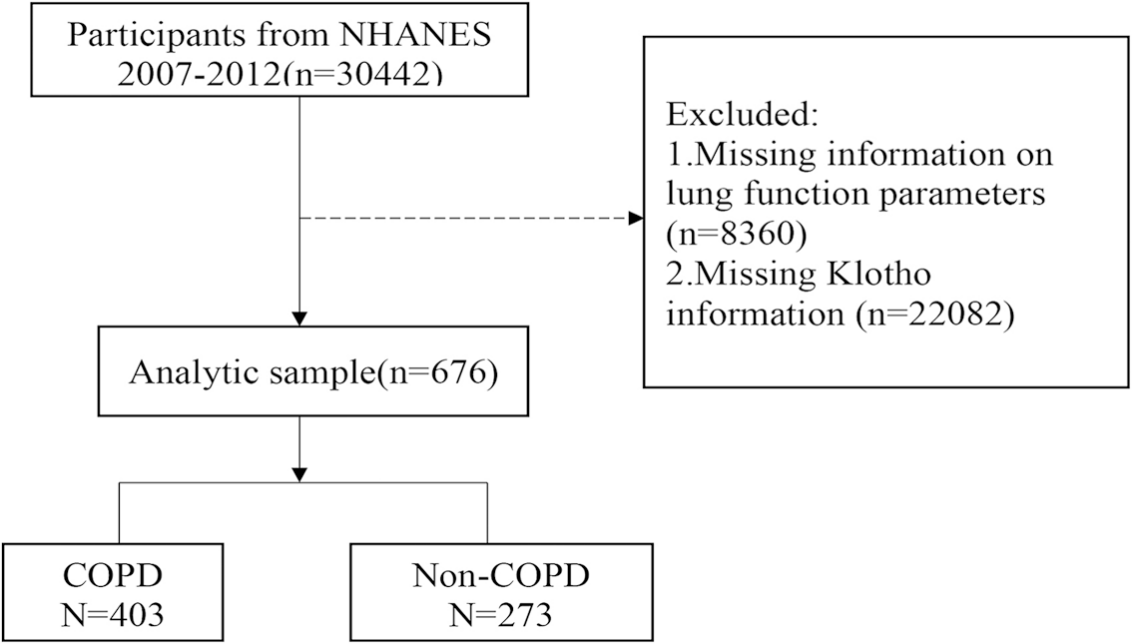
Flow of participants. COPD, chronic obstructive pulmonary disease.

The data came from the NHANES (2007–2008, 2009–2010, and 2011–2012), which is a cross-sectional investigation that collects information on the nutritional and health status of individuals in the US. We excluded patients with missing information on lung function parameters or α-klotho levels. A total of 676 eligible participants were eventually recruited and divided into a non-COPD group (*n* = 273) and a COPD group (*n* = 403; Figure 1). Because the α-klotho test was performed only for participants older than 40 years, all study participants were aged ≥40 years. The study was approved by the local (the Jinhua Central Hospital) ethics committee. All participants in the NHANES provided written informed consent. The NHANES protocol was approved by the Center’s ethics review board (24).

### Study covariates

We obtained clinical features and demographic characteristics, which included sex, body mass index [weight (kg)/height^2^ (m)], age, race (other Hispanic, Mexican American, non-Hispanic Black, non-Hispanic White, or other race), education (some college or AA degree, 9–11th grade, less than 9th grade, high-school grade, and college graduate or above), history of chronic diseases [hypertension, chronic heart disease (CHD), and diabetes], pulmonary function tests [pre- and postbronchodilator forced expiratory volume in 1 sec (FEV1), pre- and postbronchodilator forced vital capacity (FVC)], smoking status, drinking status, and laboratory parameters (α-klotho, total cholesterol, creatinine, triglycerides, albumin, leukocyte, erythrocyte, thrombocyte, and hemoglobin). All data are publicly available on the NHANES website.^1^

According to the definition of COPD, the FEV1/FVC ratio was less than 70% without reversibility (the bronchodilation effect was less than 12%). The reference values for FEV1 and the measured FEV1 relative to the predicted FEV1 (FEV1% pred) were determined using equations from the Global Lung Function Initiative 2012. Alcohol consumption was defined as consuming at least 12 alcoholic beverages of any type in any 1 year (yes/no). Three classifications of smokers were created based on the Smoking–Cigarette Use questionnaire: never smoker (smoked <100 lifetime cigarettes), former smoker (smoked ≥100-lifetime cigarettes and currently not smoking), and current smoker (smoked ≥100 lifetime cigarettes and currently smoking).

### Serum α-klotho concentrations

NHANES participants aged 40–79 had their blood drawn after fasting overnight. Until analysis, blood samples were stored at −80°C.The levels of serum α-klotho were quantified through enzyme-linked immunosorbent assay (ELISA) and then analyzed by statisticians with specialized training. Two wells were made in the ELISA plate to measure the α-klotho concentration of the quality control samples, and the average value was used as the final concentration. Before being released for reporting, all the results were reviewed to ensure that they met the laboratory’s standardized criteria for acceptability. For more information on the α-klotho detection method, we visited the NHANES website.^2^ The patients were grouped based on the tertiles of α-klotho levels (tertiles of α-klotho levels: Q1: <687 pg./mL; Q2: 687–900 pg./mL; Q3: ≥900 pg./mL).

### Statistical analysis

Descriptive statistics were used to summarize baseline characteristics, including frequencies or percentages for categorical variables and medians (interquartile ranges) or means (standard deviations) for continuous variables. To compare groups, Mann–Whitney U tests, t tests, chi-square tests, and Fisher’s exact tests were used. Logistic regression analysis was adopted to estimate the factors that influence COPD incidence. The α-klotho levels were analyzed both as a continuous and a categorical variable (tertiles of α-α-klotho levels: Q1: <687 pg./mL; Q2: 687–900 pg./mL; Q3: ≥900 pg./mL). The relationship between α-klotho levels and COPD was assessed by a generalized additive model and logistic regression analysis. For accurate estimation of associations and variances, appropriate weights were calculated according to NHANES recommendations. All COPD participants were stratified according to the levels of α-klotho, and their clinical characteristics were compared. In all analyses, R v3.5.1 was used to account for the complex survey weights, and *p* < 0.05 was used as the significance threshold.

## Results

In 59.6% (403/676) of the patients, participants were diagnosed with COPD based on their lung function. The levels of α-klotho in the COPD group were significantly lower than those in the non-COPD group (817.51 ± 302.20 vs. 863.09 ± 267.13, *p* < 0.05). Non-Hispanic White individuals were more common among COPD patients. Compared with the non-COPD population, COPD patients were older, more likely to be male, and had a greater incidence of chronic heart disease (CHD). In addition, COPD patients presented higher levels of creatinine, uric acid, erythrocytes, and hemoglobin. Among the COPD patients, the largest proportion were current smokers (38.46%), and the lowest proportion were never smokers (24.32%; Table 1).

**Table 1:** Comparisons of clinical characteristics between COPD and Non-COPD groups weighted.

Variables	Non-COPD *N* = 273	COPD *N* = 403	*p*-value
Demographic characteristic			
Age	56.93 ± 10.27	61.78 ± 10.27	<0.001
Gender (%)			<0.001
Male	146 (53.48%)	298 (73.95%)	
Female	127 (46.52%)	105 (26.05%)	
Race			<0.001
Mexican American	37 (13.55%)	21 (5.21%)	
Other Hispanic	21 (7.69%)	18 (4.47%)	
Non-Hispanic White	163 (59.71%)	280 (69.48%)	
Non-Hispanic Black	39 (14.29%)	67 (16.63%)	
Other Race	13 (4.76%)	17 (4.22%)	
Education			0.164
Less than 9th grade	32 (11.72%)	30 (7.44%)	
9-11th grade	40 (14.65%)	68 (16.87%)	
High school graduate	56 (20.51%)	106 (26.30%)	
Some college or AA degree	77 (28.21%)	108 (26.80%)	
College graduate or above	68 (24.91%)	91 (22.58%)	
BMI	27.40 ± 5.53	27.70 ± 5.36	0.482
Smoking status			<0.001
Never smokers	124 (45.42%)	98 (24.32%)	
Current smokers	85 (31.14%)	155 (38.46%)	
Former smokers	64 (23.44%)	150 (37.22%)	
Current drinking status			0.389
Drinking	47 (17.22%)	80 (19.85%)	
Non-drinking	226 (82.78%)	323 (80.15%)	
Hypertension			0.089
No	173 (63.37%)	229 (56.82%)	
Yes	100 (36.63%)	174 (43.18%)	
Diabetes			0.516
No	229 (83.88%)	345 (85.61%)	
Yes	40 (14.65%)	49 (12.16%)	
Do not know	4 (1.47%)	9 (2.23%)	
Chronic heart disease			0.032
No	264 (96.70%)	370 (91.81%)	
Yes	8 (2.93%)	27 (6.70%)	
Do not know	1 (0.37%)	6 (1.49%)	
Laboratory parameters			
Kloth Tertile(pg/ml)			0.023
<687	76 (27.84%)	149 (36.97%)	
687–900	92 (33.70%)	133 (33.00%)	
≥900	105 (38.46%)	121 (30.02%)	
Kloth(pg/ml)	863.09 ± 267.13	817.51 ± 302.20	0.044
Creatinine(mg/dl)	0.91 ± 0.25	0.98 ± 0.43	0.016
Total cholesterol (mg/dl)	199.79 ± 41.79	198.33 ± 43.37	0.661
Triglycerides(mg/dl)	155.60 ± 115.40	159.52 ± 102.88	0.644
Uric acid(mg/dl)	5.59 ± 3.07	5.83 ± 1.36	0.161
Albumin(g/l)	4.25 ± 0.29	4.23 ± 0.30	0.266
Leukocyte(10^9/L)	7.03 ± 2.35	7.12 ± 1.91	0.578
Erythrocytes(10^12/L)	4.61 ± 0.44	4.69 ± 0.49	0.029
Hemoglobin(g/dL)	14.28 ± 1.47	14.62 ± 1.40	0.003
Thrombocytes(10^9/L)	253.90 ± 76.04	243.35 ± 62.47	0.049
Lung function			
Prebronchial diastole FEV1%	0.87 ± 0.17	0.81 ± 0.20	<0.001
Postbronchial diastole FEV1%	0.91 ± 0.17	0.85 ± 0.19	<0.001

The characteristics of the 403 COPD participants according to their α-klotho levels are shown in Table 2. More COPD participants (*n* = 149) belonged to the Q1 group, indicating that low levels of α-klotho appeared to be more frequent in patients with COPD, while only 133 individuals belonged to the Q2 group and 121 individuals belonged to the Q3 group. The prebronchial diastole FEV1% pred was lower in Q1 (0.73 ± 0.10) than in Q2 (0.74 ± 0.09) and Q3 (0.75 ± 0.10). The postbronchial diastole FEV1% pred was lower in Q1 (0.77 ± 0.08) than in Q2 (0.78 ± 0.08) and Q3 (0.79 ± 0.08). Compared with individuals in the Q2 or Q3 groups, individuals in the Q1 group were more likely to have higher levels of creatinine and leukocytes, lower levels of erythrocytes, and higher proportions of current smokers and drinkers.

**Table 2:** Descriptive analysis of participants with COPD by different klotho levels weighted.

Variables	<687 *N* = 149	687–900 *N* = 133	≥900 *N* = 121	*p*-value
Demographic characteristic				
Age	62.50 ± 10.02	61.35 ± 10.72	61.37 ± 10.11	0.519
Gender (%)				0.057
Male	32 (21.48%)	32 (24.06%)	41 (33.88%)	
Female	117 (78.52%)	101 (75.94%)	80 (66.12%)	
Race				0.486
Mexican American	6 (4.03%)	7 (5.26%)	8 (6.61%)	
Other Hispanic	5 (3.36%)	4 (3.01%)	9 (7.44%)	
Non-Hispanic White	103 (69.13%)	98 (73.68%)	79 (65.29%)	
Non-Hispanic Black	29 (19.46%)	17 (12.78%)	21 (17.36%)	
Other Race	6 (4.03%)	7 (5.26%)	4 (3.31%)	
Education				0.581
Less than 9th grade	11 (7.38%)	12 (9.02%)	7 (5.79%)	
9-11th grade	31 (20.81%)	17 (12.78%)	20 (16.53%)	
High school graduate	37 (24.83%)	34 (25.56%)	35 (28.93%)	
Some college or AA degree	38 (25.50%)	42 (31.58%)	28 (23.14%)	
College graduate or above	32 (21.48%)	28 (21.05%)	31 (25.62%)	
Smoking status				0.002
Never smokers	26 (17.45%)	30 (22.56%)	42 (34.71%)	
Current smokers	72 (48.32%)	51 (38.35%)	32 (26.45%)	
Former smokers	51 (34.23%)	52 (39.10%)	47 (38.84%)	
Current drinking status				0.02
Drinking	22 (14.77%)	24 (18.05%)	34 (28.10%)	
Non-drinking	127 (85.23%)	109 (81.95%)	87 (71.90%)	
Hypertension				0.504
No	81 (54.36%)	74 (55.64%)	74 (61.16%)	
Yes	68 (45.64%)	59 (44.36%)	47 (38.84%)	
Diabetes				0.215
No	132 (88.59%)	117 (87.97%)	96 (79.34%)	
Yes	15 (10.07%)	13 (9.77%)	21 (17.36%)	
Do not know	2 (1.34%)	3 (2.26%)	4 (3.31%)	
Chronic heart disease				0.311
No	139 (93.29%)	118 (88.72%)	113 (93.39%)	
Yes	7 (4.70%)	12 (9.02%)	8 (6.61%)	
Do not know	3 (2.01%)	3 (2.26%)	0 (0.00%)	
BMI	27.32 ± 4.89	27.74 ± 5.10	28.11 ± 6.14	0.82
HEIGHT	172.91 ± 8.38	172.43 ± 9.59	169.99 ± 10.18	0.061
WEIGHT	81.98 ± 17.16	83.18 ± 20.28	81.21 ± 18.62	0.565
Laboratory parameters				
Creatinine(mg/dl)	1.06 ± 0.66	0.96 ± 0.19	0.92 ± 0.18	0.032
Total cholesterol (mg/dl)	198.99 ± 41.96	200.61 ± 46.90	194.99 ± 41.16	0.403
Triglycerides(mg/dl)	158.89 ± 102.89	157.30 ± 88.57	162.75 ± 117.27	0.709
Leukocytes(10^9/L)	7.28 ± 1.88	7.20 ± 2.05	6.83 ± 1.78	0.033
Erythrocytes(10^12/L)	4.61 ± 0.51	4.74 ± 0.45	4.70 ± 0.49	0.013
Hemoglobin(g/dL)	14.48 ± 1.29	14.78 ± 1.35	14.61 ± 1.57	0.064
Thrombocytes(10^9/L)	247.21 ± 56.78	239.52 ± 69.16	242.81 ± 61.65	0.26
Albumin(g/l)	4.21 ± 0.30	4.22 ± 0.29	4.26 ± 0.32	0.499
Lung function				
Prebronchial diastole FEV1	2518.96 ± 728.55	2547.59 ± 787.24	2365.16 ± 830.47	0.058
Prebronchial diastole FVC	4049.17 ± 1038.51	4094.00 ± 1105.99	3832.16 ± 1271.27	0.06
Postbronchial diastole FVC	4124.05 ± 1011.62	4153.58 ± 1116.05	3891.31 ± 1251.04	0.058
Postbronchial diastole FEV1	2621.22 ± 720.56	2673.92 ± 809.18	2463.15 ± 828.31	0.053
Prebronchial diastole FEV1/FVC	0.62 ± 0.07	0.62 ± 0.06	0.62 ± 0.07	0.714
Postbronchial diastole FEV1/FVC	0.63 ± 0.06	0.64 ± 0.06	0.63 ± 0.06	0.816
Prebronchial diastole FEV1% pred	0.73 ± 0.10	0.74 ± 0.09	0.75 ± 0.10	0.041
Postbronchial diastole FEV1% pred	0.77 ± 0.08	0.78 ± 0.08	0.79 ± 0.08	0.042

**Figure 2:**
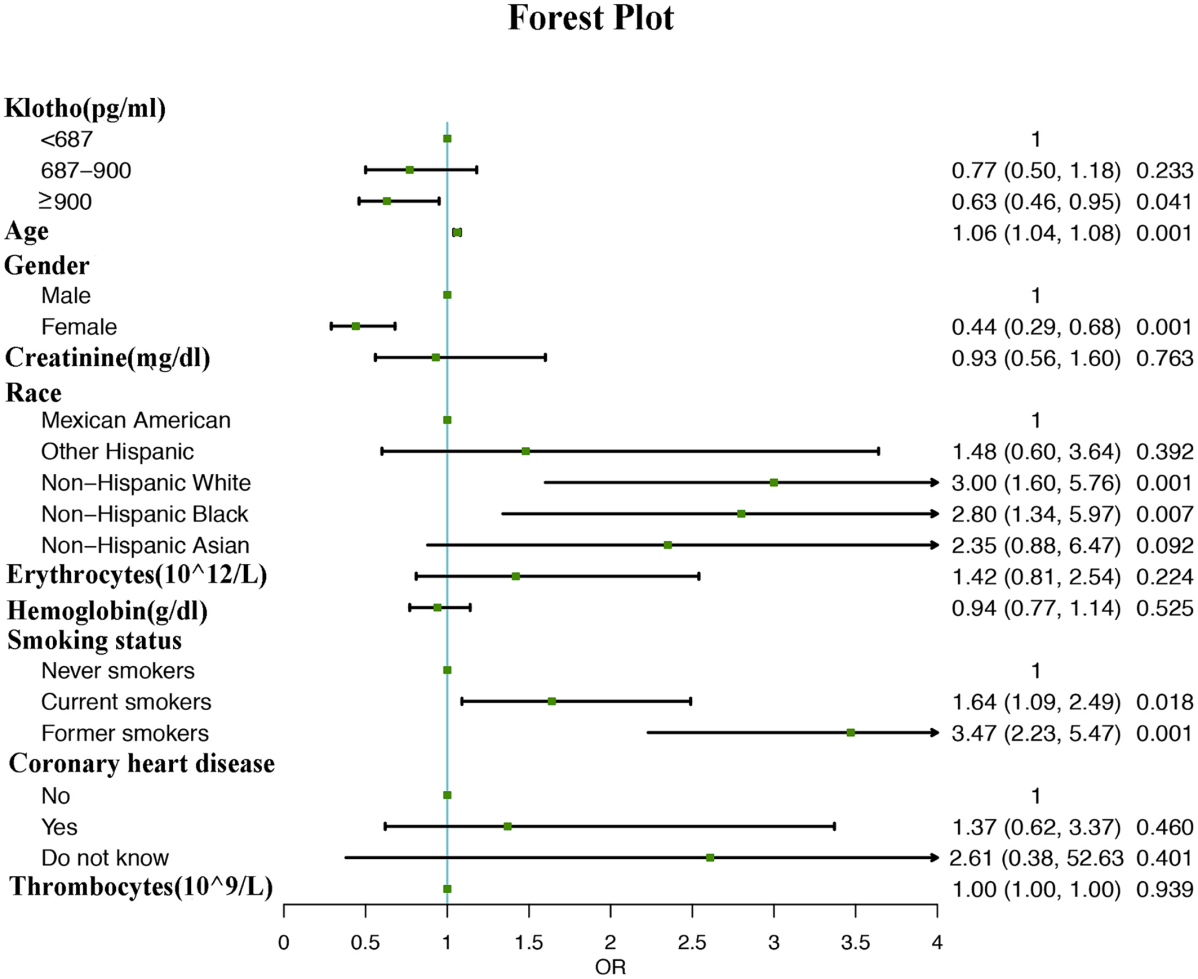
The forest plot results of the multivariate logistic regression analysis.

Multivariate logistic regression analysis revealed that participants with higher α-klotho concentrations had a lower probability of developing COPD according to both the unadjusted model (OR: 0.97; 95% CI: 0.96–0.98, *p* < 0.001) and adjusted model (Model I adjust for: age, race, smoke, gender, OR: 0.97; 95% CI: 0.96–0.99, *p* = 0.0343; model II adjust for: age, gender, creatinine, race, erythrocytes, hemoglobin, smoking status, coronary heart disease, thrombocytes, OR: 0.97; 95% CI: 0.96–0.99, *p* = 0.0348). Moreover, the association persisted when α-klotho was included in the regression model as a categorical variable. According to the unadjusted model, participants in the Q2 (OR: 0.73; 95% CI: 0.50–0.99, *p* = 0.0048) and Q3 (OR: 0.58; 95% CI: 0.41–0.86, *p* = 0.0061) α-klotho layers were less likely to develop COPD than those in the Q1 layer were. These findings remained unchanged in both adjustment Model I (Q2 vs. Q1, OR: 0.80; 95% CI: 0.52–1.21, *p* = 0.0.2880; Q3 vs. Q1, OR: 0.70; 95% CI: 0.42–0.96, *p* = 0.0313) and model II (Q2 vs. Q1, OR: 0.77; 95% CI: 0.55–1.18, *p* = 0.2330; Q3 vs. Q1, OR: 0.63; 95% CI: 0.46–0.95, *p* = 0.0410; Table 3). Moreover, the forest plot showed that smoking status, male sex, non-Hispanic white race, non-Hispanic black race and older age could be used as independent risk factors (Figure 2).

**Table 3:** Association between klotho levels and the risk of COPD among adults in NHANES 2007–2012.

	Non-adjusted OR (95%CI)	Adjust I OR (95%CI)	Adjust II OR (95%CI)
Kloth(pg/ml)	0.97 (0.96, 0.98) 0.0002	0.97 (0.96, 0.99) 0.0343	0.97 (0.96, 0.99) 0.0348
Kloth Tertile(pg/ml)			
Q1 < 687	Reference	Reference	Reference
Q2 687–900	0.73 (0.50, 0.99) 0.0048	0.80 (0.52, 1.21) 0.2880	0.77 (0.50,1.18) 0.2330
Q3 ≥ 900	0.58 (0.41, 0.86) 0.0061	0.70 (0.42, 0.96) 0.0313	0.63 (0.46,0.95) 0.0410

Model I adjust for: age, race, smoke, gender. Model II adjust for: age, gender, creatinine, race, erythrocytes, hemoglobin, smoking status, coronary heart disease, thrombocytes.

**Figure 3:**
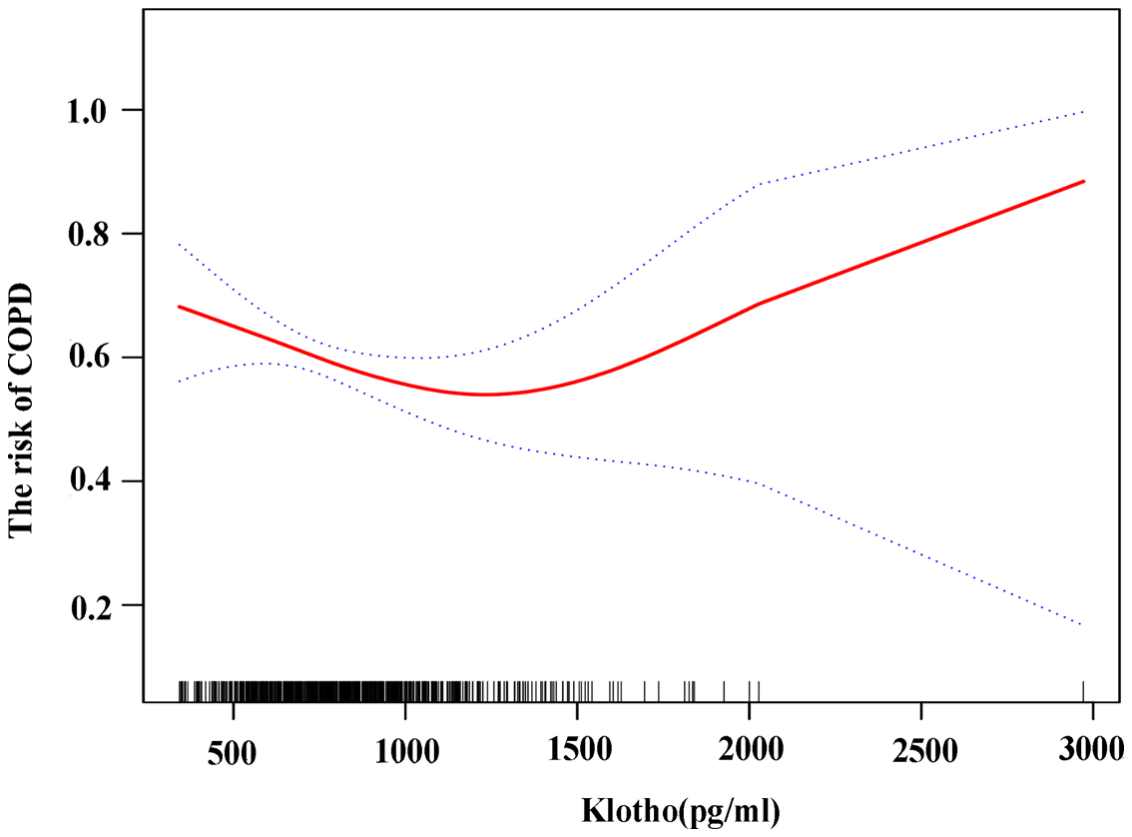
Association between klotho and risk of COPD using generalized additive model. The risk of COPD is gradually reduced as klotho levels rise.

The generalized additive model showed that α-klotho levels were negatively correlated with the risk of COPD with α-klotho levels <1,500 pg./mL. When α-klotho was ≥1,500 pg./mL, the risk of COPD increased as α-klotho levels increased. (Figure 3).

## Discussion

The main finding of this study is that the levels of α-klotho were strongly associated with the incidence of COPD in the general U.S. population. The risk of COPD gradually decreases as α-klotho levels increase with α-klotho levels <1,500 pg./mL in participants over 40 years old. When α-klotho was ≥1,500 pg./mL, the risk of COPD increased as α-klotho levels increased. In individuals with COPD, variations in pulmonary ventilation function (prebronchial diastole FEV1% pred and postbronchial diastole FEV1% pred) and hemocyte counts (leukocytes and erythrocytes) have been observed among patients with differing levels of α-klotho.

Consistent with previous studies, we found that older age, chronic cigarette smoking and male sex were risk factors for COPD (25). Additionally, our study included the anti-aging protein klotho in the analysis. Klotho is a unidirectional transmembrane protein that protects cells from reactive oxygen species and inflammation (26). Deficiencies in the klotho gene increase lifespan, while mutations cause accelerated aging and early death, supporting the idea that this gene is linked to the senescence process (27). Klotho’s anti-inflammatory and antioxidant properties play a major role in several diseases, including nephrotic syndrome (28), hypertension, heart failure (29) and lung carcinoma (30). COPD patients’ alveolar macrophages and airway epithelium showed reduced levels of klotho in previous research (17, 18). A recent study revealed that serum klotho levels were negatively correlated with COPD prevalence (23). Similar findings were obtained in this study, with reduced serum klotho levels in COPD patients. Moreover, the risk of COPD gradually decreased as the klotho concentration increased. However, MS-Patel’s study found that serum Klotho levels were not lower in COPD patients and had no link to quadriceps Klotho protein (31). Maybe it is due to small sample size.

COPD is caused by a combination of genetic and environmental factors that damage the lungs over time. There are several possible explanations for why the risk of COPD gradually decreases as α-klotho levels increase when α-klotho levels are less than 1,500 pg./mL, although the detailed underlying mechanisms are not well understood. Therefore, the first potential explanation for the pathogenesis of COPD is the influence of environmental factors such as cigarette smoking, biomass exposure, occupational exposure (including heavy metal and smoke exposure), and air pollution (comprised of particles, heavy metals, ozone, nitrogen or sulfur oxides, and other greenhouse gases) on α-klotho levels. Several studies have indicated that the serum klotho level is lower in smokers than in nonsmokers (32, 33). By measuring klotho expression in the lungs of COPD patients and in a mouse model of ozone-induced COPD, Wei Gao et al. reported that klotho expression was reduced in the lungs of smokers and further decreased in the lungs of COPD patients, and 6 weeks of ozone exposure reduced klotho levels in airway epithelial cells (17). In our study, we found that COPD individuals with COPD with low levels of klotho (<687 pg./mL) were more likely to be current smokers (*p* < 0.05). Moreover, klotho overexpression ameliorated lung damage. A study reported that a mouse tubular epithelial cell line was sensitive to the antiapoptotic and antioxidative effects of klotho (34). An *in vitro* experiment demonstrated that klotho overexpression could reduce tobacco-induced cell damage by decreasing reactive oxygen species (35). However, few studies have shown that klotho levels are elevated in individuals who smoke cigarettes (36). This may be explained by the compensatory effects on oxidative stress and inflammation in smokers. Exposure to heavy metals has been found to be associated with alterations in klotho levels. Donghoon Kim and colleagues discovered a negative correlation between blood lead and levels and serum α-klotho levels in a representative population sample of US adults (37). Cadmium exposure affects Klotho methylation levels, which may impact COPD (38–40). Another study demonstrated a negative correlation between serum α-Klotho levels and urinary thiocyanate levels, a common environmental pollutant (41). Nevertheless, there remain numerous gaps in knowledge, including the potential relationship between klotho and dust or air particles. These gaps need to be filled urgently through research. A second explanation may be the relationship between the levels of klotho and age. Age is frequently regarded as a risk factor for COPD due to the physiological deterioration in lung function that typically occurs with advancing age (42). Numerous studies have demonstrated a decrease in both the expression and circulating levels of klotho with advancing age (43–45). In our investigation, the average age of individuals with klotho levels <687 years was greater in the COPD group than in the other two groups (*p* > 0.05). However, this disparity did not reach statistical significance, potentially attributable to bias resulting from the limited sample size. Interestingly, the level of circulating klotho appears to be complex. For instance, findings from a systematic review indicated that engaging in physical activity and exercise resulted in elevated levels of circulating klotho (46). Thus, bias may result from many factors. A third explanation may be that klotho affects inflammation and oxidative stress. Chronic inflammation narrows the airways and decreases lung mobility (47). A study showed that both exogenous and endogenous klotho could enhance the capacity to fight oxidative stress in pulmonary epithelia, protecting the lung against hyperoxia- and phototoxicity-induced oxidative damage (26). Stefanie Krick et al. reported that klotho knockout mice developed COPD and exhibited airway inflammation in their lungs, whereas klotho overexpression attenuated airway inflammation (48, 49). However, there is a need for more experimental evidence to support these conjectures.

Our study demonstrated that when the concentration of klotho was greater than 1,500 pg./mL, the risk of COPD increased slightly with increasing klotho. This may be because they compensate for oxidative stress and inflammation. Some studies have shown that increased klotho might be related to the augmentation of inflammatory responses and oxidative damage. Candelaria Martín-González et al. reported that the level of klotho increased in response to increased TNF-α, a proinflammatory cytokine (50). Abdelmalik et al. reported that in septic shock disease, increased klotho levels were related to mortality (51). Alvarez-Cienfuegos et al. reported that in rheumatoid arthritis patients, the levels of klotho were high (52). Therefore, an increase in klotho was observed under certain inflammatory conditions.

In addition, our study revealed that individuals with COPD from the Q1 group, compared with those from the Q2 or Q3 groups, were more likely to have lower levels of erythrocytes and higher levels of leukocytes and creatinine (*p* < 0.05). Our results revealed a correlation between klotho and hemocyte counts (leukocytes and erythrocytes), consistent with previous research findings (53–56). Our research also revealed that individuals with COPD from the Q1 group were more likely to have lower prebronchial diastole FEV1% pred and postbronchial diastole FEV1% pred than those from the Q2 or Q3 groups. However, Haiyan Mao’s research found a positive relationship between klotho and FeNO, as well as a correlation between klotho levels and lung function (57). At present, research on the effect of klotho on pulmonary ventilation function in COPD patients is relatively rare. This association needs further investigation to verify its causality.

Our study has a few limitations. (1) Because this study was retrospective, it may have been prone to selection and information bias, and we cannot determine a causal relationship between α-klotho and COPD. It is necessary to conduct prospective and cohort studies involving a much larger population. (2) There may still be potentially confounding factors affecting the association between COPD incidence and α-klotho levels. (3) The study findings may only be generalized to subjects aged ≥40 years. (4) This study does not consider the interventions, encompassing both therapeutic and non-therapeutic measures, that are likely to influence klotho levels.

## Conclusion

Compared with those with COPD, healthy individuals retain a greater level of α-klotho. A U-shaped relationship between α-klotho and the risk of COPD was found in our study. In addition, significant differences in pulmonary ventilation function and hemocyte counts were observed among COPD patients with differing levels of α-klotho.

## Data availability statement

Publicly available datasets were analyzed in this study. This data can be found at: http://www.cdc.gov/nchs/nhanes/.

## Ethics statement

Ethical approval was not required for the study involving humans in accordance with the local legislation and institutional requirements. Written informed consent to participate in this study was not required from the participants or the participants’ legal guardians/next of kin in accordance with the national legislation and the institutional requirements.

## Author contributions

DY: Writing – original draft, Writing – review & editing.

## Footnotes

^1^ http://www.cdc.gov/nchs/nhanes/

^2^ http://www.cdc.gov/nchs/nhanes/
